# Classification of chronic pain for the *International Classification of Diseases* (ICD-11): results of the 2017 international World Health Organization field testing

**DOI:** 10.1097/j.pain.0000000000002287

**Published:** 2021-04-08

**Authors:** Antonia Barke, Beatrice Korwisi, Robert Jakob, Nenad Konstanjsek, Winfried Rief, Rolf-Detlef Treede

**Affiliations:** a Clinical and Biological Psychology, Catholic University Eichstaett-Ingolstadt, Eichstätt, Germany; b Division of Clinical Psychology, Philipps-University Marburg, Marburg, Germany; c Department of Classification and Terminologies, World Health Organization, Geneva, Switzerland; d Medical Faculty Mannheim, Mannheim University, Heidelberg, Germany

**Keywords:** ICD, Field testing, Chronic pain, Classification, Diagnosis, WHO

## Abstract

Because chronic pain has been poorly represented in the *International Statistical Classification of Diseases and Related Health Problems* (ICD) despite its significant contribution to the burden of disease worldwide, the International Association for the Study of Pain (IASP) developed a classification of chronic pain that was included in the ICD-11 version as “MG30” and approved by the *World Health Assembly* in 2019. The objective of this field test was to determine how well the classification of chronic pain works in the context of the ICD-11. A web-based survey using the WHO-FiT platform recruited 177 healthcare professionals from all WHO regions. After a training on coding chronic pain hosted by the IASP Web site, participants evaluated 18 diagnostic codes (lines) of the 2017 frozen version of the ICD-11 and 12 vignettes (cases) describing chronic pain conditions. Correctness, ambiguity, and perceived difficulty of the coding were compared between the ICD-11 and the ICD-10 and the applicability of the morbidity rules for the ICD-11 verified. In the line coding, 43.0% of correct chronic pain diagnoses assigned with the ICD-10 contrasted with 63.2% with the ICD-11. Especially in cases in which the chronic pain is regarded as the symptom of an underlying disease, the ICD-11 (63.5%) commanded more correct diagnoses than the ICD-10 (26.8%). The case coding was on average 83.9% accurate, only in 1.6% of cases any difficulty was perceived. The morbidity rules were applied correctly in 74.1% of cases. From a coding perspective, the ICD-11 is superior to the ICD-10 in every respect, offering better accuracy, difficulty, and ambiguity in coding chronic pain conditions.

## Abstract 2

Supplemental Digital Content is Available in the Text.

The official WHO field tests of the *International Classification of Diseases 11* classification of chronic pain demonstrate an excellent diagnostic coding performance and clinical utility of the new classification.

## 1. Background

Chronic pain is very frequent^7,9,13^ and one of the most common reasons to seek medical attention.^18,19^ It is a major contributor to the global burden of disease,^8,24^ leading to individual suffering and substantial direct and indirect societal costs.^2,11,14,20,27^ Yet, up to now, in the *International Classification of Diseases* *and Related Health Problems* (ICD), chronic pain diagnoses were not represented systematically.^25,26,33^ Reporting of mortality and morbidity, visibility in health statistics, consideration in public health policies, and research agendas depend on ICD diagnoses. In addition, many healthcare systems make the referral for multidisciplinary pain treatment conditional on suitable ICD codes as indicators of such health needs. The lack of appropriate codes contributes to the paucity of clearly defined treatment pathways for patients with chronic pain.

An international and multidisciplinary task force of the *International Association for the Study of Pain* (IASP) developed a systematic classification of chronic pain.^32,33^ Chronic pain is defined as pain that lasts or recurs for longer than 3 months. The classification contains 7 main diagnostic categories, distinguishing chronic primary pain^21^ and chronic secondary pain syndromes,^3,5,22,28,29^ and integrates existing pain diagnoses including headaches.^6^ The 7 main categories contain subdiagnoses to achieve a detailed classification. For every diagnosis, precise descriptions, operationalized criteria, and characteristic features are provided according to the World Health Organization (WHO) content models for the ICD-11. These pain diagnoses have been implemented in the 11^th^ version of the ICD that was released by the WHO in June 2018^38^ and approved by the World Health Assembly in May 2019.^40^ The ICD-11 will come into effect on January 1, 2022, for international health reporting.^40^

The WHO's main revision objective was improving the ICD's clinical utility.^15,23,34,35^ Clinical utility reflects the degree to which a classification system offers a conceptualization of the diagnostic entities, fosters the selection of adequate treatments, is easy and feasible to use and applicable in clinical practice, predicts prognosis and treatment outcomes, and facilitates communication and documentation.^1,12,16,17^

The ICD relies on codes to standardize the collection and communication of health data. The correct code assignment once a diagnosis has been named is of paramount importance because the assigned code forms the basis of all further information processing in the health systems. So-called morbidity rules (Box 1) govern which condition should be coded as the main condition for which health care was sought in any particular encounter if more than one health complaint is present.^39^ New diagnoses included in the ICD-11 must demonstrate that they integrate with this system and allow reliable code assignments and applications of the morbidity rules.

In this study, we aim to provide an empirical basis for including the new diagnostic categories of chronic primary and secondary pain in the ICD-11. We compare correctness and ambiguity of the new ICD-11 codes with those of the ICD-10; evaluate perceived ease of use, clinical utility, and appropriateness of coding details; and examine the compatibility with the ICD-11 morbidity rules.

## 2. Methods

### 2.1. Recruitment and participant sample characteristics

From June to August 2017, the IASP conducted one of several specialty-specific field trials parallel to the WHO field testing of the ICD-11 Mortality and Morbidity Statistics (MMS) 2017.^37^ Data of participants from Germany were simultaneously forwarded to a set of field trials commissioned by the German Ministry of Health.^31^ Healthcare professionals working with patients affected by chronic pain were invited through the IASP, as well as other medical and psychological societies; we did not recruit professional coders. Participants preregistered their email address, received a brief online training, and were invited to the WHO's ICD-FiT^10^ online portal as the platform became available. Because it was a formative field test, the testing had to be completed within the brief time window allowed by the revision process: The testing commenced with the availability of the WHO's ICD-FiT Web site and had to be closed by the latest date that the data could still inform the ICD-11 revision process. Therefore, we did not calculate an ideal sample size but strove to collect as many data sets as possible in the timeframe available.

A total of n = 177 participants (mean age 43.8 ± 11.1 years, 57.6% men, 42.4% women) from 35 countries accepted the invitation and rated a grand total of 2576 lines and 1342 cases; for comparison, the WHO field trial recruited 1673 participants from 31 countries and evaluated 112,383 code assignments.^37^ Participants in this study stemmed from all WHO regions, with the largest subsamples from Germany (20.9%), United Kingdom (13.0%), China, and Australia (9.0% each). Their professions included medicine (63.8%), physiotherapy (19.8%), clinical psychology (9.6%), dentistry (2.8%), and other (4%).

### 2.2. Material

The field testing consisted of 2 parts (line coding and case coding) and an additional section in which the participants provided a general evaluation of the new ICD-11 classification compared with the current ICD-10 classification. To familiarize the participants with the novel pain classification, they could watch training slides on the IASP Web site and received a document with training material explaining the new diagnoses and the use of the different WHO platforms (ICD-11 Browser, ICD-FiT, and the ICD-11 Coding Tool). The training lasted approximately 15 minutes, and in the end, the participants were asked to complete a 10-item self-test in a multiple choice format. If a wrong answer was chosen, the participant received the information that the answer was incorrect and the correct answer was displayed. The material was also available for download in ISO-certified translations into Chinese, English, German, Japanese, Portuguese, and Spanish. The testing itself was conducted in English with the ICD-FiT online tool (version 2.7.1).^10^

For all testing, the ICD-10 version of 2016^36^ and the ICD-11 Mortality and Morbidity Linearization was used in the frozen version of April 2, 2017 (version for quality control), to ensure a constant reference. The frozen version comprised 33 genuine chronic pain diagnoses (excluding the automatically generated categories of “other” and “unspecified”). The current 2020 frozen version (“for preparation of implementation” https://icd.who.int/browse11/l-m/en) has evolved since then, now including further chronic pain entities and a coding tool with improved search functions.

The lines and cases were prepared with the help of feedback from the experts of the IASP taskforce for the respective pain diagnoses and in cooperation with the WHO requirements. For the line coding, 18 lines reflecting the names of individual pain diagnoses as they are used in clinical practice were prepared (see Supplementary Material S1 for the list of lines, available at http://links.lww.com/PAIN/B357). For the case coding, 12 case vignettes were formulated. In each vignette, 2 or more diagnoses were present to represent different combinations of diagnoses and morbidity rules, when determining the reasons for encounter with the health system (see below). The number of lines and cases was determined on theoretical grounds: The number of lines to reflect the main categories and subcategories of chronic pain implemented in the frozen version of the browser at the time. The cases also used the main categories of chronic pain and were created to allow for combinations of categories and morbidity rules.

#### 2.2.1. Line coding

For the line coding, 18 diagnostic terms (“lines”) were selected to reflect the range of chronic pain conditions relevant for morbidity coding,^33^ and reference codes were prepared for the ICD-11 as well as for the ICD-10. For the ICD-11, reference standards were the 2017 MMS codes. For the ICD-10, only in 50% of the cases a reference standard existed; in the other cases, the “correct” diagnosis was an auxiliary way of expressing the syndrome in ICD-10. The auxiliary code consisted of a combination of etiological codes plus some code for chronic pain (R52.1 or R52.2) (cf S1, Supplementary information, available at http://links.lww.com/PAIN/B357). The auxiliary codes were allowed to give the ICD-10 the best chance despite the large number of missing diagnoses for chronic pain.^25^

For each line presented, the participant had to determine the appropriate codes in the ICD-10 (using a link to the ICD-10 browser, 2016 version) and the ICD-11 (using a link to 2017 frozen version of the ICD-11 MMS and a special coding tool). The starting point (ICD-10 or ICD-11) was randomized. After coding the lines, the participants were asked whether they had encountered any difficulty in assigning the code (*yes* or *no*); whether the level of specificity was appropriate (*not detailed enough* or *just right* or *too detailed*); and whether they had experienced any ambiguity in making the assignment (*no* or *yes*, *because …*).

#### 2.2.2. Case coding

The case coding examined the ICD-11 alone. The main objective was testing the morbidity rule application concerning the chronic pain diagnoses. For this purpose, 12 short case vignettes (mean length = 78 words, range: 50-113 words) were prepared. Each vignette featured 2 or more health conditions, at least one of which was a chronic pain condition. The vignette stated a main condition and an “other condition,” without listing the ICD-11 codes. (Table 1, vignette texts, cf supplementary information S2, available at http://links.lww.com/PAIN/B357).

**Table 1:** Chronic pain and other conditions featured in the vignettes in the case coding.

Chronic pain diagnoses	ICD-11 code*	Other condition
†Fibromyalgia	MJ60.12	Obstructive sleep apnoea syndrome
Chronic migraine	8A40.3	Recurrent major depression
‡Chronic primary visceral pain and ‡chronic widespread primary pain	MJ60.11 MJ60.12	—
Chronic cancer pain	MJ60.21	Small cell carcinoma of bronchus or lung, malignant neoplasm metastasis in bone or bone marrow, vertebral column
Chronic painful chemotherapy-induced polyneuropathy	MJ60.23	Malignant neoplasms of colon or unspecified
Chronic postsurgical pain	MJ60.32	Emphysema or unspecified
Chronic central neuropathic pain	MJ60.61	Relapsing-remitting multiple sclerosis
Chronic dental pain	MJ60.72	Pulpitis or dental caries
Chronic visceral pain from persistent inflammation	MJ60.53	Endometriosis
§Chronic musculoskeletal pain from persistent inflammation due to autoimmune disorders	MJ60.413	Rheumatoid arthritis or unspecified
‡Chronic primary musculoskeletal pain and ‡chronic migraine	MJ60.13 8A40.3	—
Chronic peripheral neuropathic pain	MJ60.62	Type 2 diabetes mellitus or diabetic polyneuropathy

* The codes displayed here are the codes as implemented in the ICD-11 in 2017, they were updated since.

† In vignette 1, MB1 was violated by preselecting “obstructive sleep apnoea” as the main condition when the vignette clearly suggested that the reason for the encounter was chronic widespread pain.

‡ in these vignettes, 2 chronic pain conditions were present, rather than one chronic pain condition and one other.

§ In vignette 10, MB2 was violated by selecting unspecified rheumatoid arthritis as main condition and chronic musculoskeletal pain from persistent inflammation due to autoimmune disorders as other condition, whereas according to MB2, chronic musculoskeletal pain from persistent inflammation due to autoimmune disorders should be the main condition because care was given for this.

In 10 of the vignettes, the main condition was specified correctly according to the morbidity rules; in 2 of the vignettes, the rules had been misapplied intentionally to test the participants' application of the rules in connection with chronic pain. In a first step, the participants had to provide the appropriate ICD-11 codes for each condition. In a second step, they had to judge whether the main condition was identified correctly (*yes* or *no*). For each diagnosis, we recorded whether they met with any difficulty in finding the code. At the end of each vignette, the participants rated the clinical utility of the new chronic pain code (0 = *not at all* to 5 = *very useful*) according to 3 questions: How useful is this classification (1) to describe this case in communications to colleagues (communication), (2) in facilitating the management of patients (management), and (3) for the collection of data, eg, for clinical or population databases (documentation)?

#### 2.2.3. General evaluation

After the participant had completed rating the lines or cases, an additional questionnaire became available in which the participant rated the overall coverage of the conditions in the ICD-11 (*5* = *very good* or *1 = very poor*), the level of detail (*not detailed enough* or *just right* or *too detailed*), and the ease of use (*5 = very easy* or *1 = very difficult*). In addition, textboxes were provided for the participants to mention whether they perceived gaps and redundancies.

### 2.3. Data analysis

#### 2.3.1. Line coding

For the ICD-10 and the ICD-11, the percentage of correct codes for each diagnosis and the percentage of difficulties with assigning the code were computed. The variable for the reported ambiguity of assigning the diagnosis was transformed into a binary variable (*yes* or *no*) and the frequencies determined. The frequencies of correctly assigned diagnoses, cases without difficulties, and cases without ambiguity were compared between ICD-10 and ICD-11 with the McNemar test. The respective frequencies for specificity (*too little detail, too much detail,* or *just right*) were calculated and compared between ICD-10 and ICD-11 with the McNemar–Bowker test. We also compared the groups of chronic primary and chronic secondary pain regarding correctly assigned diagnoses, difficulties, ambiguity, and specificity.

#### 2.3.2. Case coding

The percentage of correct codes per pain diagnosis was calculated, and the mean judgements of the 3 facets of clinical utility for each pain diagnosis were computed. The correct selection of the main condition according to the morbidity rules was analysed between the rules by the χ^2^ test.

#### 2.3.3. Evaluation

Frequencies for coverage, ease of use, and level of detail were calculated. The textboxes were scrutinized for relevant comments and coded whether the participant reported gaps (*no comment* or *no gap* or *gap*) or redundancies (*no comment* or *no redundancy* or *redundancy*) and frequencies reported.

## 3. Results

### 3.1. Line coding

In total, the participants rated 2576 lines. They assigned more codes correctly using the ICD-11 (63.3%) than the ICD-10 (42.1%) (χ^2^ [1, N = 2576] = 229.23, *P* < 0.001), encountered fewer difficulties assigning ICD-11 diagnoses (no difficulty 86.6%) than ICD-10 diagnoses (47.2%) (χ^2^ [1, N = 2576] = 863.81, *P* < 0.001), and perceived fewer cases of ambiguity for the ICD-11 (no ambiguity: 75.5%) than for the ICD-10 (29.1%) (χ^2^ [1, N = 2512] = 1003.84, *P* < 0.001).

**Figure 1.**
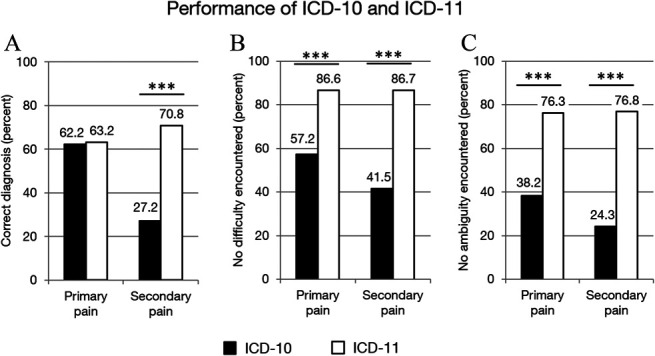
Performance of the ICD-10 and the ICD-11 in the line coding of chronic pain conditions. Correctness (A), difficulty (B), and ambiguity (C) of coding diagnostic terms of chronic primary pain conditions using the ICD-10 (filled bars) and the ICD-11 (open bars). Frequencies are shown in percent of all lines. Statistical testing with the McNemar test. ***p < 0.001.

Six of the lines referred to what is called “chronic primary pain” in the ICD-11 and 12 referred to chronic secondary pain. The diagnostic accuracy did not differ significantly in the ICD-11 between chronic primary (62.2%) and secondary pain syndromes (63,8%) (χ^2^ [1, N = 2576] = 0.64, *P* > 0.4) (Fig. 1). In the ICD-10, however, coding of chronic secondary pain was poor (27.2%) while existing chronic primary pain conditions were recognized correctly (70.8%) (χ^2^ [1, N = 2576] = 722.51, *P* < 0.001). Regarding the perceived difficulty of the code assignment, primary and secondary pain did not differ in the ICD-11 (χ^2^ [1, N = 2576] = 0.004, *P* > 0.9), but did so in ICD-10 (χ^2^ [1, N = 2576] = 58.87, *P* < 0.001), with secondary pain being more difficult (refer to Fig. 1 for percentages). A parallel picture is revealed for ambiguity of code assignment. In the ICD-11, no difference between primary and secondary pain was observed (χ^2^ [1, N = 2576] = 0.09, *P* > 0.7), but in the ICD-10, more ambiguity was reported for code assignments for secondary pain (χ^2^ [1, N = 2576] = 49.14, *P* < 0.001).

**Figure 2.**
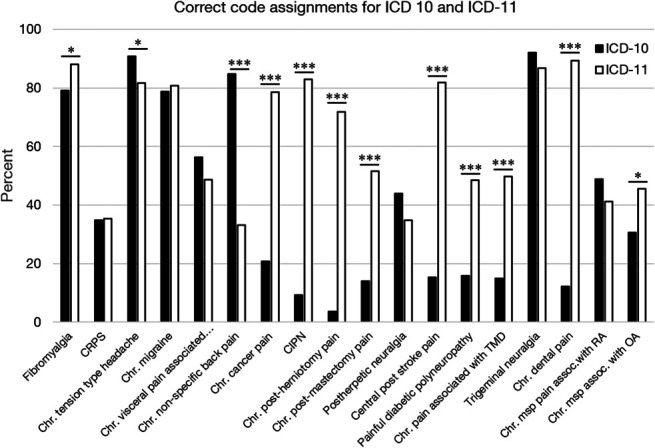
Correct code assignments per line for the ICD-10 and the ICD-11. Correct code assignments in the ICD-10 (filled bars) and the ICD-11 (open bars). Frequencies are shown as percent of the respective condition. assoc, associated; Chr, chronic; CIPN, chronic painful chemotherapy-induced polyneuropathy; CRPS, complex regional pain syndrome; IBS, irritable bowel syndrome; msp, musculoskeletal; OA, osteoarthritis; RA, rheumatoid arthritis; TMD, temporomandibular disorder. Statistical testing with the McNemar test. *P < 0.05; ***P < 0.001.

The differences in performance of the ICD-10 and the ICD-11 were also significant for most individual lines (refer to Fig. 2 for correctness; supplementary material S3-4 for difficulty and ambiguity, available at http://links.lww.com/PAIN/B357).

**Figure 3.**
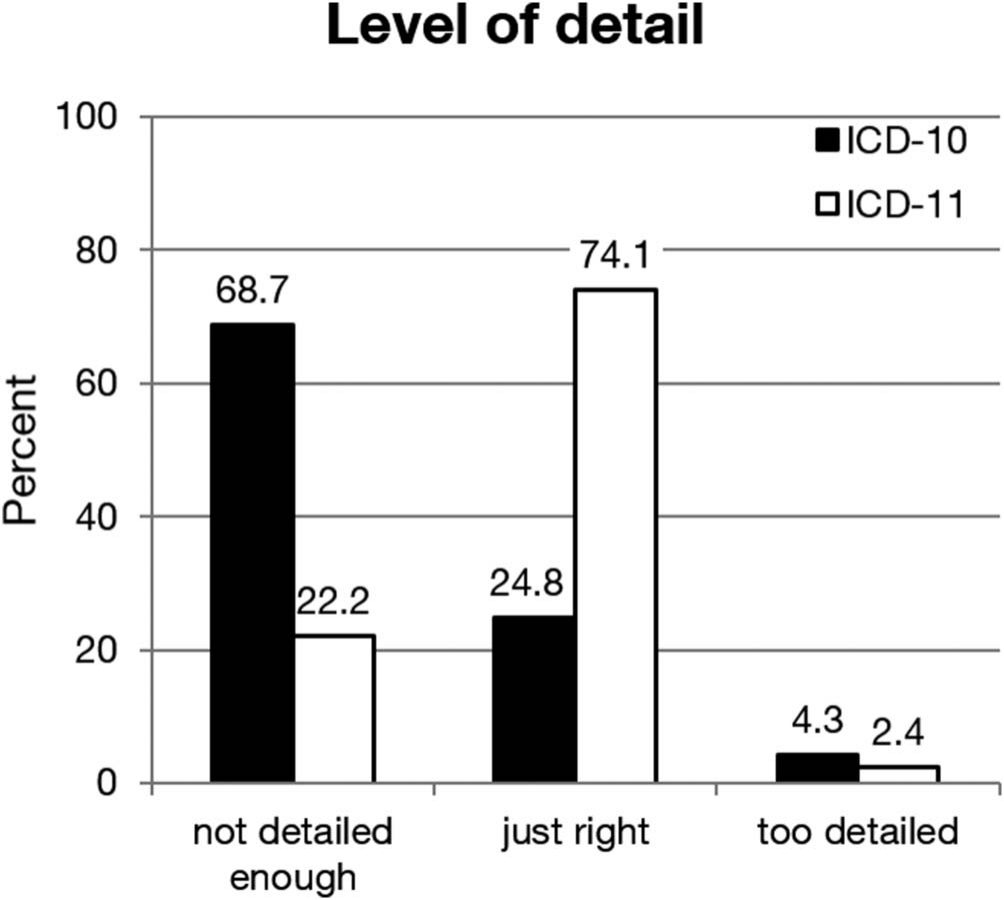
Level of specificity of the codes in the ICD-10 and the ICD-11 across all lines.

Regarding the level of detail provided in the classification, the participants' ratings favoured the ICD-11 over the ICD-10 for every diagnostic code assessed. A majority (74.1%) judged the level of detail in the ICD-11 codes as “just right” vs 24.8% for the ICD-10. (χ^2^ [3, N = 2515] = 1073.01, *P* < 0.001). Most participants regarded the level of detail in ICD-10 (68.7%) as “too low” (Fig. 3). Some lines in ICD-11 were perceived as allowing too little coding details (for a line-by-line analysis cf S5); > 30% regarded the codes for chronic nonspecific back pain, postherniotomy and postmastectomy pain, and painful diabetic neuropathy and central poststroke pain as too broad.

### 3.2. Case coding

One of the prepared cases referred to a diagnosis (Chronic peripheral neuropathic pain) that had not yet been implemented in the ICD-11 at the time of the field trial, and therefore was excluded from evaluation, leaving 13 pain diagnoses for 11 cases to be rated per participant. Taken together, the participants rated 1342 cases, each case was rated by between 67% (119/177) and 75% (132/177) of participants. Participants assigned the correct code for the pain diagnosis in 83.9% of cases, and in 98.4% of cases, participants reported having no difficulty in assigning the diagnosis (Table 2). The aggregated clinical utility (the mean for the individual facets) was high across all cases (4.3 ± 0.90) on a scale ranging from 0 (*not at all useful*) to 5 (*very useful*) and all single cases (Table 2).

**Table 2:** Case coding.

	Correct pain diagnosis (%)	No difficulty assigning pain diagnosis (%)	Aggregated clinical utility		Valid n
			Mean	SD	
All (without*)	83.9	98.4	4.3	0.90	1342
Fibromyalgia	87.1	97.7	4.2	0.93	132
Chronic migraine	51.2	97.6	4.4	0.79	127
Chronic primary visceral pain	81.6	95.2	4.2	1.09	125
Chronic cancer pain	86.0	98.3	4.3	0.97	121
CIPN	92.6	99.2	4.3	0.97	121
Chronic postsurgical pain	89.2	97.5	4.3	0.88	120
Chronic central neuropathic pain	95.0	100.0	4.4	0.77	120
Chronic dental pain	87.4	99.2	4.4	0.81	119
Chronic visceral pain from persistent inflammation	89.1	100.0	4.2	0.93	119
Rheumatoid arthritis	75.6	100.0	4.4	0.75	119
Chronic primary musculoskeletal pain	89.9	98.3	4.3	0.92	119
*Chronic peripheral neuropathic pain	—	—	—	—	

The numbers of participants (in percent) who chose the correct ICD-11 pain diagnosis and numbers of participants (in percent) who did not report any difficulty in assigning the pain diagnosis and mean ratings of clinical utility for each diagnosis.

* For chronic peripheral neuropathic pain, the correct code was not our code and no crosslinks had been implemented in the frozen version of the browser at time of the study.

CIPN, chemotherapy-induced painful polyneuropathy.

Text box 1. **Morbidity coding rules**

Morbidity rule 1 (MB1): Several conditions recorded as the “main condition.”
  Select the “main condition” for which the patient received care. Extension codes may be used to indicate different types of main condition (eg, reason for admission or main resource condition). In cases where the main condition cannot be determined based on documentation, select the condition that is mentioned first.
Morbidity rule 2 (MB2): Condition recorded as “main condition” is presenting symptom of diagnosed, treated condition.
  If a symptom or sign (ICD-11 chapter 21) or a problem (ICD-11 chapter 24) is recorded as the “main condition,” and this is obviously a sign, symptom, or problem of a diagnosed condition coded elsewhere, and care was given to the latter, reselect the diagnosed condition as the “main condition.”
Morbidity rule 3 (MB3): Signs and symptoms.
  When a symptom or sign is documented as the “main condition,” and it is documented that it could be caused by either one condition or another, select the symptom or sign as “main condition.”

**Figure 4.**
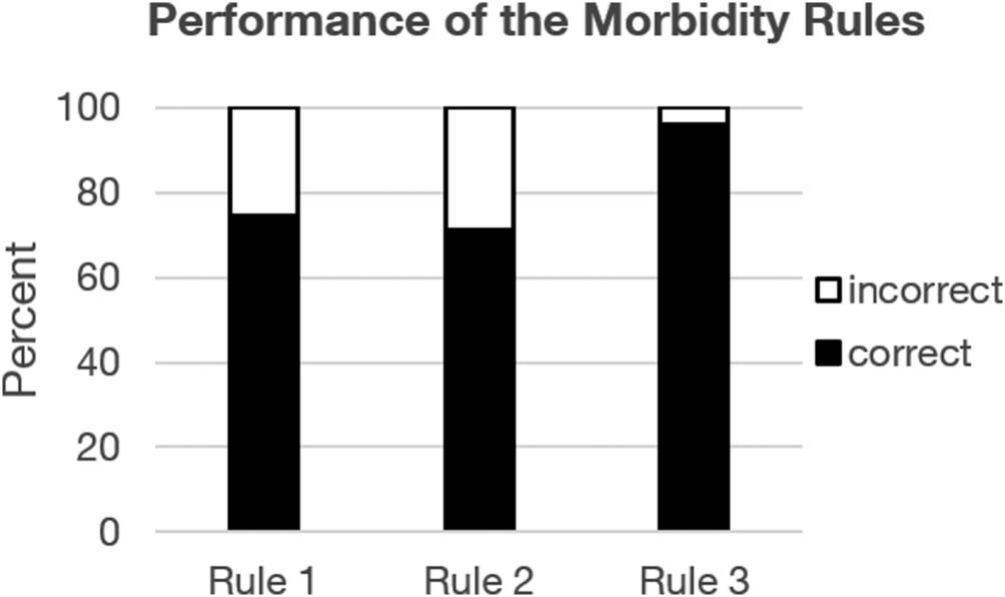
Performance of the morbidity rules with the chronic pain conditions. Frequencies of correct (filled bars) and incorrect (open bars) applications of the rules. Frequencies are shown in percent of the application of each rule.

Overall, the morbidity rules for the selection of the main condition for encounter with the health system were applied correctly in 74.1% of all cases, irrespective of whether the rule was violated in the case vignette (ie, the wrong condition cited as the reason for the encounter) or not (χ^2^ [1,N = 1461] = 3.67, *P* > 0.05). Rule 1 (74.4% correct) and 2 (71.3% correct) did not differ from each other (χ^2^ [1, N = 1187] = 1.50, *P* > 0.2), but differed from rule 3 (96.0% correct) (rule 1 vs 3: χ^2^ [1, N = 832] = 48.51, *P* < 0.001; rule 2 vs 3: χ^2^ [1, N = 807] = 58.50, *P* < 0.001) (Fig. 4).

### 3.3. General evaluation

**Figure 5.**
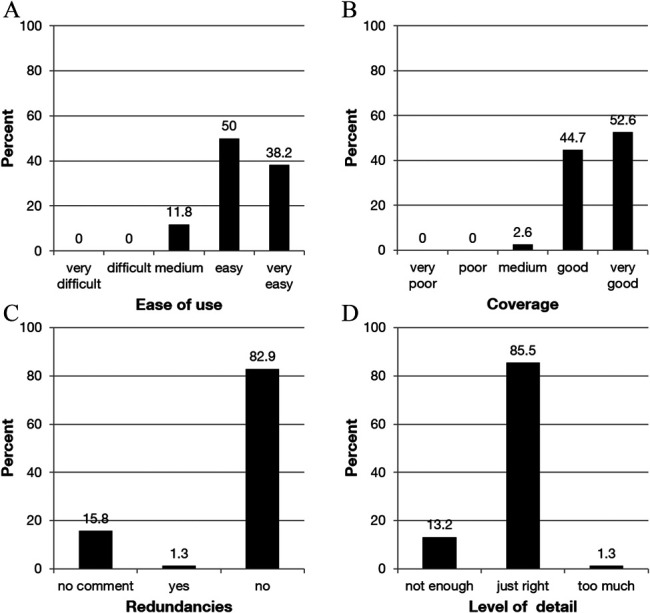
Global evaluation of the section on chronic pain in the ICD-11. Participants' ratings of ease of use (A), coverage (B), redundancies (C), and level of detail of the diagnoses (D) in the ICD-11.

In the general evaluation, 88.2% of the participants rated the ICD-11 classification of chronic pain as easy or very easy to use (Fig. 5A). Regarding the classification itself, 97.3% of the participants rated the coverage as good or very good (Fig. 5B). They perceived no redundancies (82.9%) (Fig. 5C) and rated it as having just the right level of detail (85.5%) (Fig. 5D).

In cases where participants indicated gaps, they referred mainly to chronic postsurgical or posttraumatic pain and chronic neuropathic pain.

## 4. Discussion

With this study, we supply comprehensive data of an evaluative field test demonstrating that the new chronic pain diagnoses conform to the WHO requirements for ICD-11 coding.

In the line coding, the new ICD-11 classification was tested against the established system of the ICD-10 and outperformed it in almost every way: With the ICD-11, coding was more accurate, easier, and less ambiguous to use than the ICD-10. In particular, chronic secondary pain was difficult to code in the ICD-10 (72.8% false). The lack of unique codes for these conditions in the ICD-10 required the co-selection of unspecific codes (R52) to indicate the presence of chronic pain. Most participants failed to use this code combination and offered a broad variety of codes and combinations instead, demonstrating that reliable coding of chronic pain conditions with the ICD-10 is difficult. This difficulty is reflected by the fact that these codes are rarely used in healthcare statistics. Here, the ICD-11 fared significantly better.

For 6 of the 9 lines with existing reference codes in the ICD-10, no performance difference was found between the ICD-10 and the ICD-11. Of the remaining 3 lines, fibromyalgia was coded more accurately in the ICD-11, whereas chronic nonspecific back pain and tension-type headache were coded more accurately in the ICD-10. Especially in the case of chronic nonspecific back pain, this can be attributed to the use of the ICD-11 frozen version, which did not penetrate to chronic primary low back pain, but required that the participants select chronic primary musculoskeletal pain. Although the concept of chronic primary pain is new,^21,33^ the codes were correctly identified in both, the ICD-10 and the ICD-11. However, perceived difficulty and ambiguity were less in the ICD-11 for both chronic primary and secondary pain conditions. This is particularly remarkable because the participants were more experienced coding with the ICD-10 than the ICD-11 where they received a brief online training only. With 63.3%, the absolute percentage of correctly assigned ICD-11 codes is not ideal. It represents a lower limit achieved by participants who received the briefest of trainings, had not used the new classification of chronic pain in their clinical practice, and conducted the coding in English—often not their native language. It is reasonable to expect significant improvements with thorough training, familiarity, and the use of the coders' native language once the ICD-11 is implemented and becomes the standard coding system.

To sum up, by demonstrating a reliable translation of diagnoses into codes, the chronic pain section in the ICD-11 conforms to the requirements of the WHO and promises substantial improvements over the ICD-10.

The case coding tested the selection of appropriate ICD-11 codes in the context of vignettes that provided meaningful clinical information. The code assignments were very accurate (83.9% correct), and only 1.6% reported difficulty assigning the diagnosis. This excellent performance was obtained from clinicians, not professional coders. The case coding demonstrated that chronic primary and secondary pain allows a correct rule-based selection of the main condition that was the reason for the healthcare episode. This is very encouraging as the morbidity rules were only introduced with the ICD-11 so that they were unfamiliar to the participants, who had only received a brief explanation in the training. Further improvements can be expected when the chronic pain classification is available to a more fine-grained level than it was in the frozen version.

The participating pain specialists perceived the clinical utility of the new classification as high and agreed with the utility judgements obtained in the formative field testing.^4^

The participants also indicated that the ICD-11 classification of chronic pain offers a good coverage of chronic pain syndromes, again corroborating the results from the informal field testing.^4^ The majority believed the level of detail to be appropriate and reported few gaps and very few redundancies. The main areas in which a need for more detailed diagnoses was perceived were chronic neuropathic pain and chronic postsurgical or posttraumatic pain. Here, the frozen version meant that the coding reached only down to the levels of chronic postsurgical or posttraumatic pain, or chronic central or chronic peripheral neuropathic pain. This does not reflect the classification^28,29^ but the granularity of the frozen version. At present, this is still the level to which the codes of the classification are visible in the ICD-11, although the level below, allowing for the relevant detail, has been included as index terms (“inclusion terms”) and has been assigned uniform resource identifiers.

To sum up, the field test demonstrated that the classification of chronic pain in the ICD-11 improves clinical utility and thus fulfills a central aim of the ICD revision process^15,35^: The ICD-11 was easier to use and less ambiguous than the ICD-10 and reflected all relevant categories of chronic pain without redundancy. The descriptions in the foundation were published in a series of articles (for review see Ref. 32) meeting the WHO's aim of scientific evidence for the diagnoses entered. This predicts that not only in the field of pain medicine but also for chronic pain cases in primary care the ICD-11 will be easy to implement in clinical practice.^30^ Codes were easier to find in the ICD-11 browser than the ICD-10 browser. The excellent mapping of chronic pain syndromes onto clinically meaningful codes will improve the healthcare statistics and epidemiological studies and contribute to an improved visibility of chronic pain. This, in turn is expected to lead to research programs and advances in access to treatment benefitting millions of patients with chronic pain.

### 4.1. Limitations

The new chronic pain classification allows for more detailed coding than had been implemented in the frozen version at the time. Further limitations were the complex registration process for the WHO-FiT and the short timeline for the completion of the field testing, limiting the number of participants; nonetheless, all WHO regions and relevant healthcare professions were represented in the field test. The language of this field test was English, rendering it harder for participants who were not native speakers. However, this does not invalidate the results, as one would expect factors such as ease of use of the coding system and achieved accuracy to increase when using one's native language.

## 5. Conclusions

In conclusion, the ICD-11 is superior to the ICD-10 and equally suitable for coding chronic primary and chronic secondary pain. According to results of this field test, the ICD-11 will be more precise and less ambiguous in representing chronic pain conditions in healthcare statistics in the future.

## Conflict of interest

A. Barke's position was funded by a grant from the International Association for the Study of Pain to Marburg University. The IASP did not play a role in study design, data collection, analysis and interpretation of the data, or writing the manuscript. R.-D. Treede reports grants from European Union IMI; grants from TEVA; personal fees from Bayer, Grünenthal, GSK, and Sanofi; and grants from Deutsche Forschungsgemeinschaft, outside the submitted work. The remaining authors have no conflicts of interest to declare.

## Appendix A. Supplemental digital content

Supplemental digital content associated with this article can be found online at http://links.lww.com/PAIN/B357.

## References

[1] Aasvang EK Brandsborg B Christensen B Jensen TS Kehlet H. Neurophysiological characterization of postherniotomy pain. PAIN 2008;137:173–81. 17976914 10.1016/j.pain.2007.09.026

[2] Azevedo LF Altamiro CP Medonca L Dias CC Castro-Lopes JM. The economic impact of chronic pain: a nationwide population-based cost-of-illness study in Portugal. Eur J Health Econ 2016;17:87–98. 25416319 10.1007/s10198-014-0659-4

[3] Aziz Q Giamberardino MA Barke A Korwisi B Baranowski AP Wesselmann U Rief W Treede RD, IASP Taskforce for the Classification of Chronic Pain. The IASP classification of chronic pain for ICD-11: chronic secondary visceral pain. PAIN 2019;160:69–76. 30586073 10.1097/j.pain.0000000000001362

[4] Barke A Korwisi B Casser HR Fors EI Geber C Schug S Stubhaug A Ushida T Wetterling T Rief W Treede RD. Pilot field testing of the chronic pain classification for ICD-11: the results of ecological coding. BMC Public Health 2018;180:1239. 10.1186/s12889-018-6135-9 PMC6223095 30404594

[5] Bennett MI Kaasa S Barke A Korwisi B Rief W Treede R-D, IASP Taskforce for the Classification of Chronic Pain. The IASP classification of chronic pain for ICD-11: chronic cancer-related pain. PAIN 2019;160:38–44. 30586069 10.1097/j.pain.0000000000001363

[6] Benoliel R Svensson P Evers S Wang S-J Barke A Korwisi B Rief W Treede RD, IASP Taskforce for the Classification of Chronic Pain. The IASP classification of chronic pain for ICD-11: chronic secondary headache or orofacial pain. PAIN 2019;160:60–8. 30586072 10.1097/j.pain.0000000000001435

[7] Breivik H Collett B Ventafridda V Cohen R Gallacher D. Survey of chronic pain in Europe: prevalence, impact on daily life, and treatment. Eur J Pain 2006;10:287–333. 16095934 10.1016/j.ejpain.2005.06.009

[8] Briggs AM Woolf AD Dreinhöfer K Homb N Hoy DG Kopansky-Giles D Akesson K March L. Reducing the global burden of musculoskeletal conditions. Bull World Health Organ 2018;96:366–8. 29875522 10.2471/BLT.17.204891 PMC5985424

[9] Croft P Blyth FM van der Windt D. The global occurrence of chronic pain: an introduction. In: Croft P Blyth FM van der Windt D, editors. Chronic pain epidemiology: from aetiology to public health. Oxford: Oxford University Press, 2010. pp. 9–18.

[10] Donada M Kostanjsek N Della Mea V Celik C Jakob R. Piloting a collaborative web-based system for testing ICD-11. Stud Health Technol Inform 2017;235:466–70. 28423836

[11] Dureja GP Jain PN Shetty N Mandal SP Prabhoo R Joshi M Goswami S Natarajan KB Iyer R Tanna DD Ghosh P Saxena A Kadhe G Phansalkar AA. Prevalence of chronic pain, impact on daily life, and treatment practices in India. Pain Pract 2014;14:51–62. 10.1111/papr.12132 24304963

[12] First MB Pincus HA Levine JB Williams JBW Üstün BT Peele R. Clinical utility as a criterion for revising psychiatric diagnoses. Am J Psychiatry 2004;161:946–54. 15169680 10.1176/appi.ajp.161.6.946

[13] Goldberg DS Summer JM. Pain as a global public health priority. BMC Public Health 2011:770. 21978149 10.1186/1471-2458-11-770 PMC3201926

[14] Institute of Medicine. Relieving pain in America: A blueprint for transforming prevention, care, education, and research. Washington, DC: The National Academies Press, 2011. 22553896

[15] Jakob R. [ICD-11-Adapting ICD to the 21st century]. Bundesgesundheitsblatt Gesundheitsforschung Gesundheitsschutz 2018;61:771–7. 29869704 10.1007/s00103-018-2755-6

[16] Keeley JW Reed GM Roberts MC Evans SC Medina-Mora ME Robles R Rebello T Sharan P Gureje O First MB Andrews HF Ayuso-Mateos JL Gaebel W Zielasek J Saxena S. Developing a science of clinical utility in diagnostic classification systems:field study strategies for ICD-11mental and behavioural disorders. Am Psychol 2016;71:3–16. 26766762 10.1037/a0039972

[17] Kendell R Jablensky A. Distinguishing between the validity and utility of psychiatric diagnoses. Am J Psychiatry 2003;160:4–12. 12505793 10.1176/appi.ajp.160.1.4

[18] Koleva D. Pain in primary care: an Italian survey. Eur J Public Health 2005;15:475–9. 16150816 10.1093/eurpub/cki033

[19] Mäntyselkä P Kumpusalo E Ahonen R Kumpusalo A Kauhanen J Viinamäki H Halonen P Takala J. Pain as a reason to visit the doctor: a study in Finnish primary health care. PAIN 2001;89:175–80. 11166473 10.1016/s0304-3959(00)00361-4

[20] Mayer S Spickschen J Stein VK Crevenna R Dorner TE Simon J. The societal costs of chronic pain and its determinants: the case of Austria. PLoS One 2019:1–18. 10.1371/journal.pone.0213889 PMC6426226 30893370

[21] Nicholas M Vlaeyen JWS Rief W Barke A Aziz Q Benoliel R Cohen M Evers S Giamberardino MA Goebel A Korwisi B Perrot S Svensson P Wang SJ Treede RD; IASP Taskforce for the Classification of Chronic Pain. The IASP classification of chronic pain for ICD-11: chronic primary pain. PAIN 2019;160:28–37. 30586068 10.1097/j.pain.0000000000001390

[22] Perrot S Cohen M Barke A Korwisi B Rief W Treede RD; IASP Taskforce for the Classification of Chronic Pain. The IASP classification of chronic pain for ICD-11: chronic secondary musculoskeletal pain. PAIN 2019;160:77–82. 30586074 10.1097/j.pain.0000000000001389

[23] Reed GM. Toward ICD-11: improving the clinical utility of WHO's international classification of mental disorders. Prof Psychol Res Pract 2010;41:457–64.

[24] Rice ASC Smith BH Blyth FM. Pain and the global burden of disease. PAIN 2016;157:791–6. 26670465 10.1097/j.pain.0000000000000454

[25] Rief W Kaasa S Jensen R Perrot S Vlaeyen JWS Treede RD Vissers KC. The need to revise pain diagnoses in ICD-11. PAIN 2010;149:169–70. 20346590 10.1016/j.pain.2010.03.006

[26] Rief W Kaasa S Jensen R Perrot S Vlaeyen JWS Treede R-D Vissers KCP. New proposals for the International Classification of Diseases-11 revision of pain diagnoses. J Pain 2012;13:305–16. 22480770 10.1016/j.jpain.2012.01.004

[27] Ruhe AK Wager J Linder R Meusch A Pfenning I Zernikow B. Chronischer Schmerz bei Kindern und Jugendlichen: eine ökonomische Betrachtung. Der Schmerz 2020:133–9. 32020302 10.1007/s00482-020-00446-0

[28] Scholz J Finnerup NB Attal N Aziz Q Baron R Bennett MI Benoliel R Cohen M Cruccu G Davis KD Evers S First MB Giamberardino MA Hansson P Kaasa S Korwisi B Kosek E Lavand'homme P Nicholas M Nurmikko T Perrot S Raja SN Rice ASC Rowbotham MC Schug S Simpson DM Smith BH Svensson P Vlaeyen JWS Wang SJ Barke A Rief W Treede R-D, Classification Committee of the Neuropathic Pain Special Interest G. The IASP classification of chronic pain for ICD-11: chronic neuropathic pain. PAIN 2019;160:53–9. 30586071 10.1097/j.pain.0000000000001365 PMC6310153

[29] Schug SA Lavand'homme P Barke A Korwisi B Rief W Treede RD; IASP Taskforce for the Classification of Chronic Pain. The IASP classification of chronic pain for ICD-11: chronic postsurgical or posttraumatic pain. PAIN 2019;160:45–52. 30586070 10.1097/j.pain.0000000000001413

[30] Smith BH Fors EA Korwisi B Barke A Cameron P Colvin L Richardson C Rief W Treede RD; IASP Taskforce for the Classification of Chronic Pain. The IASP classification of chronic pain for ICD-11: applicability in primary care. PAIN 2019;160:83–7. 30586075 10.1097/j.pain.0000000000001360

[31] Stausberg J Pollex-Kruger A Semler SC Vogel U Reinecke H. [Field tests for the beta version of the ICD-11-MMS in Germany: background and methods]. Bundesgesundheitsblatt Gesundheitsforschung Gesundheitsschutz 2018;61:836–44. 29845303 10.1007/s00103-018-2751-x

[32] Treede R-D Rief W Barke A Aziz Q Bennett MI Benoliel R Cohen M Evers S Finnerup NB First MB Giamberardino MA Kaasa S Korwisi B Kosek E Lavand'homme P Nicholas M Perrot S Scholz J Schug S Smith BH Svensson P Vlaeyen JWS Wang SJ. Chronic pain as a symptom or a disease: the IASP classification of chronic pain for the international classification of diseases (ICD-11). PAIN 2019;160:19–27. 30586067 10.1097/j.pain.0000000000001384

[33] Treede RD Rief W Barke A Aziz Q Bennett MI Benoliel R Cohen M Evers S Finnerup NB First MB Giamberardino MA Kaasa S Kosek E Lavand'homme P Nicholas M Perrot S Scholz J Schug S Smith BH Svensson P Vlaeyen JWS Wang SJ. A classification of chronic pain for ICD-11. PAIN 2015;156:1003–7. 25844555 10.1097/j.pain.0000000000000160 PMC4450869

[34] Ustün BT Jakob R. Calling a spade a spade: meaningful definitions of health conditions. Bull World Health Organ 2005;83:802. 16302029 PMC2626455

[35] Üstün TB Jakob R Çelik C Lewalle P Kostanjsek N Renahan M Madden R Greenberg M Chute C Virtanen M Hyman S Harrison J Ayme S Sugano K. Production of ICD-11: The overall revision process. Vol. 16, 2007. Available at: https://www.who.int/classifications/icd/ICDRevision.pdf. Accessed 26 April 2021.

[36] World Health Organisation. International statistical classification of diseases and related health problems, 10th Revision, 2016 version, Vol. 2020, 2016. Available at: https://icd.who.int/browse10/2016/en. Accessed 26 April 2021.

[37] World Health Organisation. Field-Testing ICD-11 MMS, 2017. Available at: www.who.int/classifications/2017_10_ICD11_Newsletter.pdf. Accessed 26 April 2021.

[38] World Health Organisation. Frozen version of ICD11 for implementation. 2018. Available at: http://www.who.int/classifications/icd/en/. Accessed 26 April 2021.

[39] World Health Organisation. ICD-11: Implementation or Transition Guide. Geneva: WHO, 2019. Available at: https://icd.who.int/docs/ICD-11%20Implementation%20or%20Transition%20Guide_v105.pdf. Accessed 26 April 2021.

[40] World Health Organisation. World Health Assembly Update, 2019. Available at: https://www.who.int/news/item/25-05-2019-world-health-assembly-update. Accessed 26 April 2021.

